# Supplementary material for: Recruitment of BRD4 to the ASXL1 genomic targets depends on the extra-terminal domain of BRD4
Source: Nat Commun. 2026 Feb 17;17:2852. doi: 10.1038/s41467-026-69565-z (PMC13021927; doi:10.1038/s41467-026-69565-z)
Supplement: Supplementary file 1 — Supplementary Info [file 41467_2026_69565_MOESM1_ESM.pdf]

Supplementary Information

Recruitment of BRD4 to the ASXL1 genomic targets depends on the extra-terminal domain of BRD4

Karthik Selvam, et al.

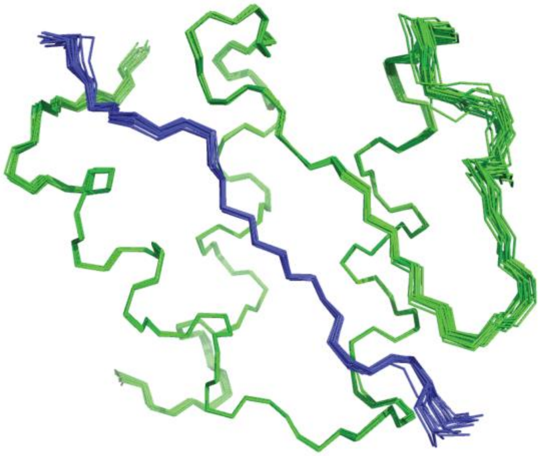

Supplementary Figure 1. A stereo view of the structure of the $BRD4_{ET}$-$ASXL1_{EBM}$ complex. Related to Figure 2.

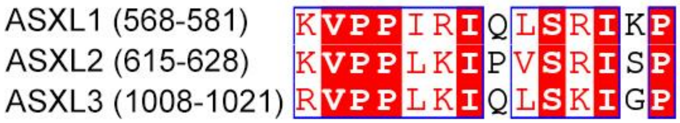

Supplementary Figure 2. Sequence alignment of the ASXL1$_{EBM}$ peptides. Related to Figure 3.

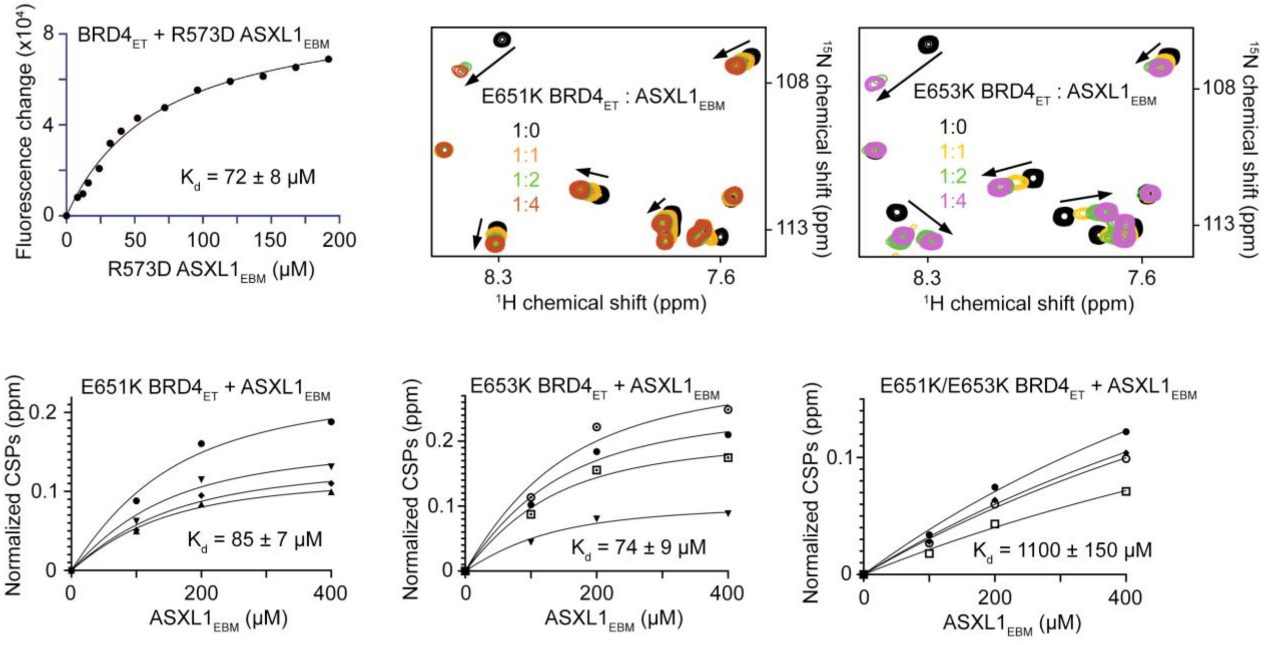

Supplementary Figure 3. Mutational analysis of the BRD4-ASXL1 interface. (top left) Representative binding curve used to determine binding affinity of wild-type $BRD4_{ET}$ to indicated mutated $ASXL1_{EBM}$ peptide by fluorescence spectroscopy. The K_d value represents average of three independent experiments, and error represents standard deviation between these experiments. (top middle and top right) Overlaid $^{1}H,^{15}N$ HSQC spectra of mutated $BRD4_{ET}$ collected upon titration with wild-type $ASXL1_{EBM}$ peptide. Spectra are color coded according to the protein:peptide molar ratio. (bottom) Binding curves used to determine binding affinities of mutated $BRD4_{ET}$ to $ASXL1_{EBM}$ peptide by NMR. The K_d value represents average of four measurements, and error represents standard deviation between these measurements. Related to Figure 4.

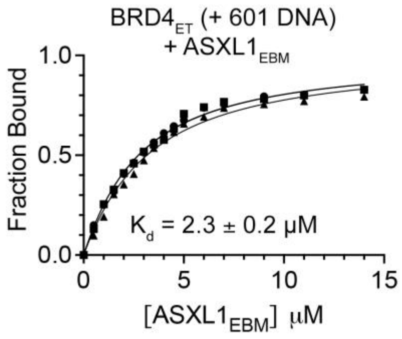

Supplementary Figure 4. ASXL1$_{EBM}$- and DNA-binding sites of BRD4$_{ET}$ do not overlap. Binding curves used to determine binding affinities of BRD4$_{ET}$ for the ASXL1$_{EBM}$ peptide in the presence of 75 nM of 147 bp 601 DNA measured by fluorescence spectroscopy. The K_d value represents average of three independent experiments, and error represents standard deviation between these experiments. Related to Figure 4.

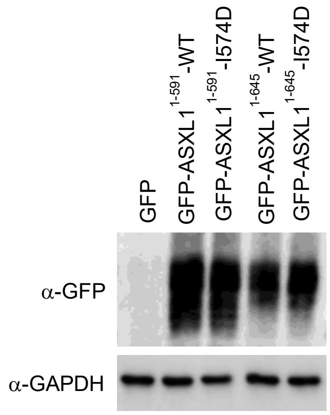

Supplementary Figure 5. Expression of the GFP-tagged ASXL1 constructs. HEK293T cells were transduced with lenti-virus expressing GFP, GFP-tagged wild-type ASXL1$^{1-591}$ and ASXL1$^{1-645}$, or GFP-tagged I581D mutants of ASXL1$^{1-591}$ and ASXL1$^{1-645}$. Whole cell lysates were immunoprecipitated using GFP-trap beads. The protein levels of the GFP-tagged ASXL1 constructs were determined by western blot using anti-GFP antibody. GAPDH, loading control. Related to Figure 5. Source data are provided as a Source Data file.

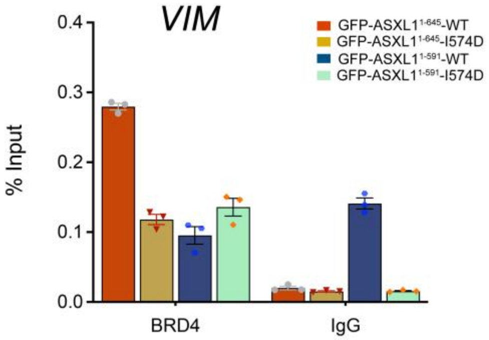

Supplementary Figure 6. ChIP-qPCR was performed in HEK293T cells transduced with GFP-ASXL1$^{1-645}$-WT or GFP-ASXL1$^{1-645}$-I574D plasmids. An antibody against BRD4 was used for ChIP. PCR was conducted with primers specific for the *VIM* gene promoter. Data represent the mean ± SEM of three independent experiments. Related to Figure 6.

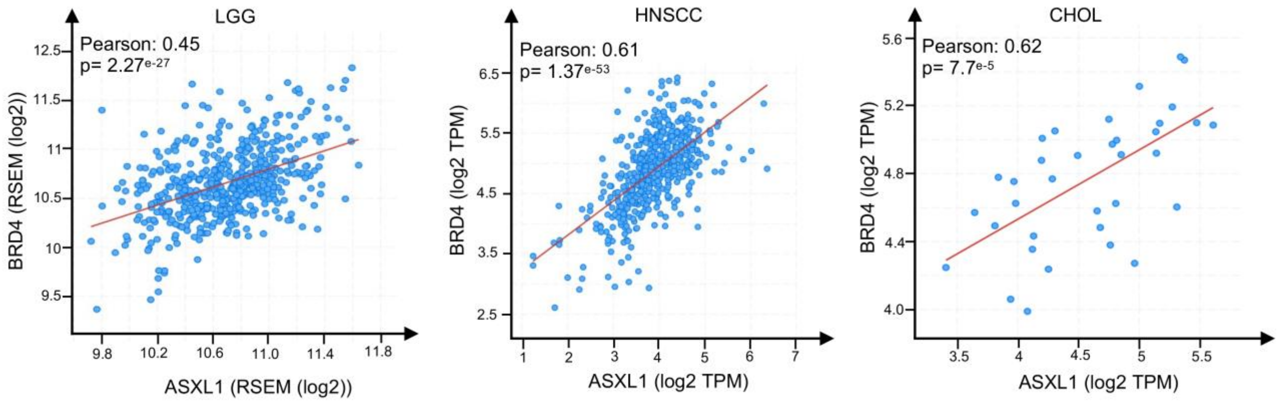

Supplementary Figure 7. Correlation analysis of BRD4 and ASXL1 mRNA across various cancer types. LGG: Brain Lower Grade Glioblastoma, HNSCC: Head and Neck Squamous Cell Carcinoma, CHOL: Cholangiocarcinoma. Related to Figure 8.

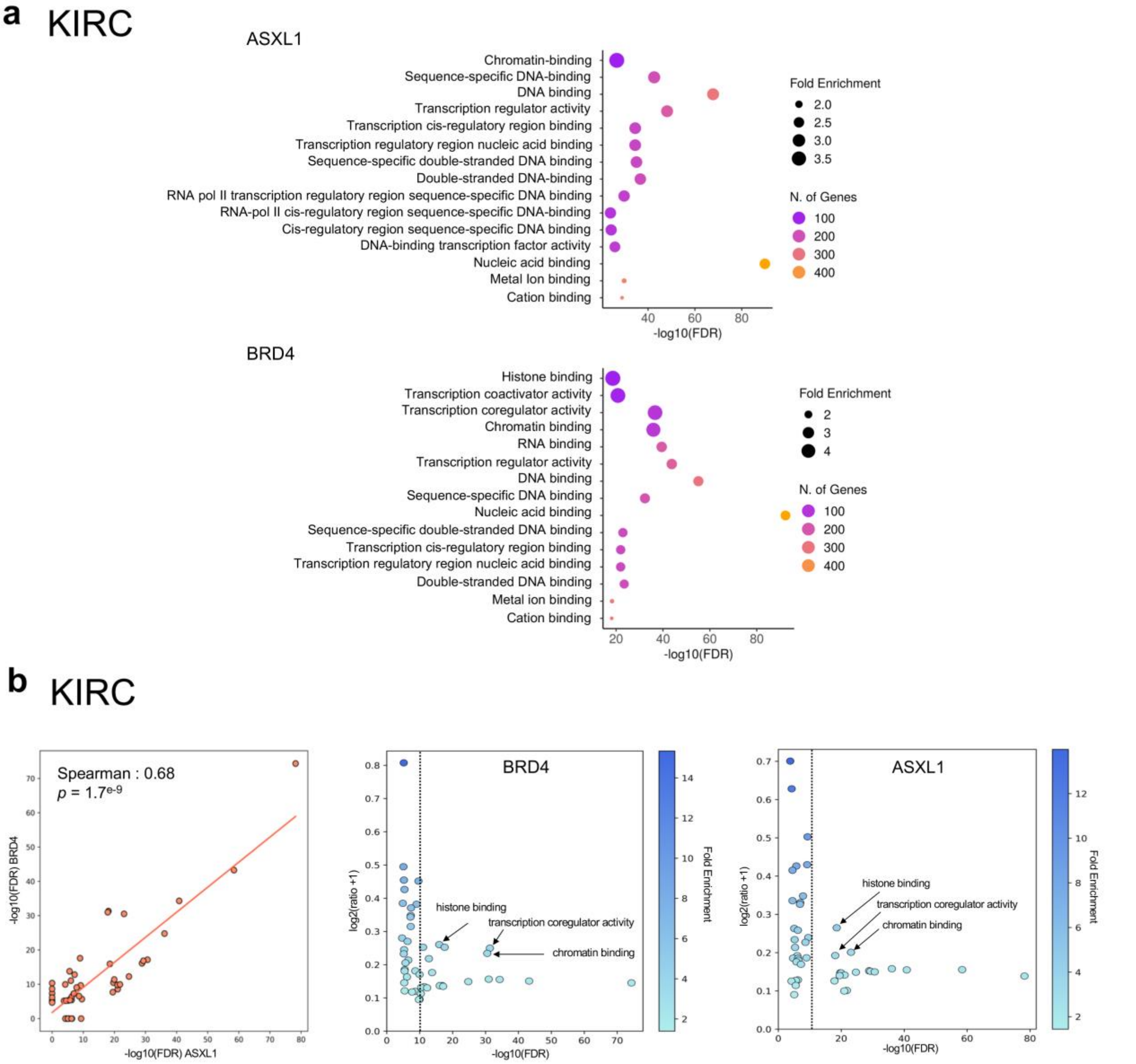

Supplementary Figure 8. Molecular Functions Enriched in ASXL1 and BRD4 highest expressing KIRC patients. (a) Gene Ontology (GO) enrichment analysis of the top 1,000 genes upregulated in the top quartile of ASXL1- or BRD4- expressing KIRC patients. GO terms are ranked by statistical significance ($-\log_{10}$ FDR). Dot size reflects the Fold enrichment while color indicates number of genes annotated to each term. (b) Scatterplot showing correlation of GO Molecular Functions pathway significance between ASXL1 and BRD4 in a KIRC cohort (left). Scatterplot of GO enrichment metrics for ASXL1 and BRD4 (middle and right). Related to Figure 8.

Supplementary Table 1. Summary of restraints and statistics of the final 20 structures of $BRD4_{ET}$ in complex with ASXL1 peptide. Related to Figure 2.

Conformationally restricting restraints	
Total distance restraints	1972
Intra-residue	745
Sequential (|i - j| = 1)	341
Medium-range (1 < |i – j| < 5)	363
Long-range (|i - j| ≥ 5)	207
Inter-chain	217
Hydrogen bond restraints	99
Total dihedral angle restraints	154
Phi angle	77
Psi angle	77
Ramachandran Map Analysis (%)[a]	
Most favored regions	96.4
Additional allowed regions	3.6
Generally allowed regions	0.0
Disallowed regions	0.0
Structure statistics	
Violations (mean +/- s.d.)	
Distance constraints (Å)	0.052 +/- 0.0061
Dihedral angle constraints (º)	0.46 +/- 0.081q
Max. dihedral angle violation (º)	0.71
Max. distance constraint violation (Å)	0.064
Deviations from idealized geometry	
Bond lengths (Å)	0.0051+/- 0.00012
Bond angles (º)	0.64 +/- 0.013
Impropers (º)	1.6 +/- 0.063
Average pairwise RMSD calculation (Å)[b,c]	
Heavy	0.58 +/- 0.051
Backbone	0.22 +/- 0.048

[a]: Procheck calculation was done for protein residues 608-640, 649-654, 659-676 of BRD4 and 569-574 of ASXL1.

[b]: The residue number ranges used in full molecule pairwise root-mean-square deviation (RMSD) calculations consists of 608-676 of BRD4 and 568-576 of ASXL1.

[c]: Pairwise RMSD was calculated among top 20/200 lowest energy structures.
